# A High Red Blood Cell Distribution Width Predicts Failure of Arteriovenous Fistula

**DOI:** 10.1371/journal.pone.0036482

**Published:** 2012-05-04

**Authors:** Krzysztof Bojakowski, Mensur Dzabic, Ewa Kurzejamska, Grzegorz Styczynski, Piotr Andziak, Zbigniew Gaciong, Cecilia Söderberg-Nauclér, Piotr Religa

**Affiliations:** 1 Department of General, Vascular and Oncologic Surgery, Warsaw University of Medicine, Warsaw, Poland; 2 Department of Medicine, Center for Molecular Medicine, Karolinska Institutet, Stockholm, Sweden; 3 Department of Internal Medicine and Hypertension, Warsaw University of Medicine, Warsaw, Poland; Institut national de la santé et de la recherche médicale (INSERM), France

## Abstract

In hemodialysis patients, a native arteriovenous fistula (AVF) is the preferred form of permanent vascular access. Despite recent improvements, vascular access dysfunction remains an important cause of morbidity in these patients. In this prospective observational cohort study, we evaluated potential risk factors for native AVF dysfunction. We included 68 patients with chronic renal disease stage 5 eligible for AVF construction at the Department of General and Vascular Surgery, Central Clinical Hospital Ministry of Internal Affairs, Warsaw, Poland. Patient characteristics and biochemical parameters associated with increased risk for AVF failure were identified using Cox proportional hazards models. Vessel biopsies were analyzed for inflammatory cells and potential associations with biochemical parameters. In multivariable analysis, independent predictors of AVF dysfunction were the number of white blood cells (hazard ratio [HR] 1.67; 95% confidence interval [CI] 1.24 to 2.25; *p*<0.001), monocyte number (HR 0.02; 95% CI 0.00 to 0.21; *p* = 0.001), and red blood cell distribution width (RDW) (HR 1.44; 95% CI 1.17 to 1.78; *p*<0.001). RDW was the only significant factor in receiver operating characteristic curve analysis (area under the curve 0.644; CI 0.51 to 0.76; *p* = 0.046). RDW>16.2% was associated with a significantly reduced AVF patency frequency 24 months after surgery. Immunohistochemical analysis revealed CD45-positive cells in the artery/vein of 39% of patients and CD68-positive cells in 37%. Patients with CD68-positive cells in the vessels had significantly higher white blood cell count. We conclude that RDW, a readily available laboratory value, is a novel prognostic marker for AVF failure. Further studies are warranted to establish the mechanistic link between high RDW and AVF failure.

## Introduction

In hemodialysis patients, a native arteriovenous fistula (AVF) is the preferred form of permanent vascular access [1]. Complications associated with vascular access for hemodialysis are important causes of morbidity among end-stage renal disease patients [2]. In 2% to 53% of patients, AVFs are never usable for dialysis (primary failures), owing to early thrombosis or failure to mature [3]. The major cause of late AVF failure is venous stenosis and subsequent thrombosis due to progressive neointimal hyperplasia [4]. The thrombosis-free patency rate in patients with an autogenous AVF is 42% to 90% at 12 months [3], [5].

Risk factors for AVF thrombosis include diabetes mellitus, anticardiolipin antibodies, patient age, previous use of a dialysis catheter, hypoalbuminemia, and high serum levels of lipoprotein A and fibronectin. Patients with diabetes mellitus have elevated serum coagulation factors and impaired fibrinolysis due to elevated concentrations of plasminogen activator inhibitor-1 (PAI-1), resulting in a higher frequency of atherothrombotic events as well as venous thrombosis [6]–[8]. High anti-Cytomegalovirus IgG levels have also been identified as an independent risk factor for AVF thrombosis [9], [10] but we were unable to confirm these findings [11].

Patients with end-stage renal disease have a bleeding diathesis caused by a reduced number of platelets and alterations in platelet adhesion and aggregation. Paradoxically, these patients are also predisposed to thrombosis due to impaired endothelial function and elevations in PAI-1, homocysteine, and von Willebrand factor [12]. Prothrombotic alternations in coagulation factors, such as increases in fibrinogen, thromboplastin, and fibrinopeptide A levels and in the activity of factors VII, VIII, and IX-XII, have also been demonstrated in patients with renal failure [12].

Persistent inflammation has also been identified as a risk for AVF thrombosis. Serum C reactive protein (CRP) levels greater than 0.8 mg/dl conferred an 16.6 times increased risk for vascular access thrombosis in patients with native AVF [13]. Elevations in other inflammatory factors such as IL-6 and PAI-1 have also been associated with AVF failure [14].

Although numerous risk factors and correlates to AVF dysfunction have been identified, they do not explain all cases of vascular access thrombosis. In this prospective cohort study, we evaluated the predictive value of numerous biochemical factors, assessed at the time of surgery, for AVF failure.

## Materials and Methods

### Ethics statement

The study was approved by the Ethical Committee of the Central Clinical Hospital Ministry of Internal Affairs (40/2005) and the Ethical Committee at Karolinska Institutet (2011/411-31/1). Written informed consent was obtained from all patients.

### Study design and patients

Patients with stage 5 chronic kidney disease who had an expected survival time >12 months and qualified for primary AVF construction were included in the study. The exclusion criteria were previous surgery of the forearm, advanced heart failure (New York Heart Association class III or IV), or obstruction of the cephalic vein or radial artery before surgery. Between June 1, 2006, and September 30, 2007, 68 patients with established chronic kidney disease received a primary AVF between the radial artery and cephalic vein at the Department of General and Vascular Surgery Central Clinical Hospital Ministry of Internal Affairs and were included in the study. The patients were followed for 24 months after surgery. The primary outcome measure was AVF failure.

At 12 and 52 weeks after surgery and in cases of dysfunction, AVF patency was evaluated by ultrasonography to measure fistula blood flow and cephalic vein diameter and to identify arterial or venous stenosis. AVFs with signs of pathology were evaluated with angiography before AVF failure was diagnosed. AVF dysfunction was defined as failure of the AVF to mature or thrombosis or stenosis that decreased blood flow and resulted in inadequate dialysis.

### Analysis of blood and biochemical factors

Blood samples were taken at the day of surgery and evaluated for the factors shown in Tables 1 and 2 using routine methods at the Central Clinical Hospital of Ministry of Internal Affairs. Blood parameters were analyzed with an automated hematology analyzer (Sysmex XT 2000, GMI Inc.). Biochemical factors were analyzed with a Cobas Integra 800 system (Roche); hemostasis factors were analyzed with a BCS XP System (Siemens). Albumin-corrected calcium was calculated as total serum calcium (mg/dL)+0.0704×(34 – serum albumin (g/L)).

**Table 1:** Patient Characteristics.

Patient characteristics	Early AVF dysfunction (n = 11)	Late AVF dysfunction (n = 23)	No AVF dysfunction (n = 34)	*p*	All patients (n = 68)
Mean age (years, ±SD)	68±12	60±15	61±16	0.33	62±15
Gender [n (%)]				0.87	
Male	7 (63.6)	15 (65.2)	24 (70.6)		46 (67.6)
Female	4 (36.4)	8 (34.8)	10 (29.4)		22 (32.4)
Smoker [n (%)]	4 (36.4)	13 (56.5)	16 (47.1)	0.53	33 (48.5)
Other diseases [n (%)]					
Heart failure	7 (70.0)	18 (81.8)	28 (84.8)	0.45	53 (81.5)
Diabetes	2 (22.2)	12 (54.5)	8 (25.8)	0.07	22 (35.5)
Hyperlipidemia	3 (33.3)	3 (13.6)	8 (25.8)	0.41	14 (22.6)
Hypertension	7 (70.0)	18 (81.8)	28 (84.8)	0.57	53 (81.5)
Medications [n (%)]					
Antithrombotic agents	2 (20.0)	8 (34.8)	12 (40.0)	0.52	22 (34.9)
Anticoagulative agents	1 (11.1)	0 (0.0)	1 (3.3)	0.28	2 (3.2)
β blockers	4 (40.0)	18 (78.3)	23 (74.2)	0.07	45 (70.3)
Calcium antagonists	4 (40.0)	15 (65.2)	24 (77.4)	0.09	43 (67.2)
ACEI	3 (30.0)	11 (47.8)	14 (45.2)	0.62	28 (43.8)
Statins	2 (22.2)	9 (39.1)	15 (48.4)	0.36	26 (41.3)
Biochemical parameters					
C-reactive protein (mg/L, ± SD)	18.6±16.8	8.6±7.7	7.3±6.6	**0.01**	9.2±9.3
Procalcitonin (ng/L, ± SD)	0.24±0.08	0.19±0.07	0.21±0.06	0.24	0.21±0.07
HDL cholesterol (mg/dL, ± SD)	52.1±18.5	50.8±18.0	56.7±17.6	0.54	54.2±17.7
LDL cholesterol (mg/dL, ± SD)	108.6±48.1	104.5±53.1	99.5±45.7	0.89	102.4±47.7
Triglyceride (mg/dL, ± SD)	158.4±75.8	152.4±97.1	148.6±82.4	0.96	151.1±85.2
Creatinine (mg/dL, ± SD)	5.2±1.9	4.6±1.4	5.1±1.9	0.74	5.1±1.8
Albumin (g/dL, ± SD)	3.2±1.1	3.6±0.6	4.0±0.4	**0.003**	3.8±0.7
Albumin corrected calcium (mg/L, ± SD)	8.7±0.6	8.2±0.7	8.6±0.4	0.09	8.5±0.6
White blood cell count (10^9^ cells/L, ± SD)	9.3±2.3	7.2±1.9	7.1±1.7	**0.01**	7.4±2.0
Neutrophils (10^9^ cells/L, ± SD)	6.2±2.6	4.8±1.6	4.6±1.2	0.06	4.9±1.6
Lymphocytes (10^9^ cells/L, ± SD)	1.6±0.3	1.8±0.8	1.6±0.7	0.41	1.7±0.7
Monocytes (10^9^ cells/L, ± SD)	0.7±0.2	0.6±0.2	0.7±0.3	0.22	0.7±0.3
Hematocrit (%, ± SD)	28.7±3.0	30.8±3.6	32.3±4.5	0.07	31.3±4.1
Red blood cells (×10^12^/L, ± SD)	3.2±0.3	3.5±0.4	3.7±0.6	**0.048**	3.5±0.5
Hemoglobin (g/dL, ± SD)	9.7±1.0	10.4±1.4	10.9±1.5	0.08	10.6±1.5
MCV (fL, ± SD)	89.4±2.6	89.3±7.7	89.1±4.7	0.99	89.2±5.8
MCH (pg, ± SD)	30.1±1.4	30.2±3.1	30.4±2.0	0.93	30.3±2.4
RDW (%, ± SD)	16.9±2.4	15.6±2.3	14.8±1.7	**0.03**	15.4±2.1
Platelets (×10^3^/µL, ± SD)	329±143	231±80	257±89	**0.049**	257±98
Iron (µmol/L, ± SD)	10.9±3.9	11.5±5.7	11.5±6.9	0.96	11.4±6.1
Transferrin (g/L, ± SD)	1.8±0.3	1.9±0.5	1.8±0.5	0.77	1.8±0.4
Ferritin (µg/L, ± SD)	170.4±104.7	189.3±202.6	235.7±314.5	0.69	209.4±254.1

Boldface indicates statistically significant values (p<0.05). ACEI, angiotensin-converting enzyme inhibitor; HDL, high-density lipoprotein; LDL, low-density lipoprotein; MCV, mean corpuscular volume; MCH, mean corpuscular hemoglobin; RDW, red blood cell distribution width.

**Table 2:** Results of Univariable and Multivariable Analyses.

Patient characteristics	Univariable analysis HR (95% CI)	*p*	Multivariable analysis	*p*
Age (y)	1.01 (0.98 to 1.03)	0.517		
Male	0.90 (0.45 to 1.82)	0.780		
Smoking	1.03 (0.53 to 2.01)	0.934		
Other diseases				
Heart failure	1.46 (0.74 to 2.88)	0.280		
Diabetes	1.58 (0.78 to 3.19)	0.207		
Hyperlipidemia	0.85 (0.35 to 2.06)	0.716		
Hypertension	0.65 (0.28 to 1.51)	0.323		
Medications				
Antithrombotic agents	0.74 (0.35 to 1.56)	0.433		
Anticoagulative agents	1.23 (0.17 to 8.95)	0.838		
β blockers	0.68 (0.33 to 1.40)	0.300		
Calcium antagonists	0.52 (0.26 to 1.03)	0.061		
ACEI	0.88 (0.44 to 1.75)	0.722		
Statins	0.67 (0.32 to 1.38)	0.278		
Biochemical parameters				
C-reactive protein (mg/L, ± SD)	1.04 (1.00 to 1.08)	**0.040**	1.00 (0.95 to 1.05)	0.895
Procalcitonin (ng/L, ± SD)	0.23 (0.00 to 65.00)	0.609		
HDL cholesterol (mg/dL, ± SD)	0.99 (0.96 to 1.01)	0.367		
LDL cholesterol (mg/dL, ± SD)	1.00 (0.99 to 1.01)	0.637		
Triglyceride (mg/dL, ± SD)	1.00 (1.00 to 1.01)	0.893		
Creatinine (mg/dL, ± SD)	0.99 (0.81 to 1.21)	0.913		
Albumin (g/dL, ± SD)	0.43 (0.26 to 0.71)	**0.001**	0.61 (0.32 to 1.16)	0.137
Albumin corrected calcium (mg/L, ± SD)	0.60 (0.30 to 1.19)	0.145	0.50 (0.22 to 1.12)	0.094
White blood cell count (10^9^ cells/L, ± SD)	1.22 (1.00 to 1.49)	**0.048**	1.67 (1.24 to 2.25)	**<0.001**
Neutrophils (10^9^ cells/L ± SD)	1.31 (0.99 to 1.72)	0.056		
Lymphocytes (10^9^ cells/L ± SD)	1.17 (0.71 to 1.93)	0.553		
Monocytes (10^9^ cells/L ± SD)	0.31 (0.07 to 1.43)	0.040	0.02 (0.00 to 0.21)	**0.001**
Hematocrit (%, ± SD)	0.92 (0.84 to 1.00)	0.062		
Red blood cells (×10^12^/L, ± SD)	0.46 (0.22 to 0.96)	**0.040**	0.98 (0.25 to 3.89)	0.973
Hemoglobin (g/dL, ± SD)	0.79 (0.61 to 1.01)	0.066		
MCV (fL, ± SD)	1.01 (0.94 to 1.07)	0.866		
MCH (pg, ± SD)	0.96 (0.83 to 1.12)	0.645		
RDW (%, ± SD)	1.18 (1.02 to 1.37)	**0.029**	1.44 (1.17 to 1.78)	**<0.001**
Platelets (×10^3^/µL, ± SD)	1.00 (1.00 to 1.00)	0.843		
Iron (µmol/L, ± SD)	0.99 (0.94 to 1.05)	0.849		
Transferrin (g/L, ± SD)	1.03 (0.48 to 2.19)	0.947		
Ferritin (µg/L, ± SD)	1.00 (1.00 to 1.00)	0.516		

Boldface indicates statistically significant values (p<0.05). ACEI, angiotensin-converting enzyme inhibitor; HDL, high-density lipoprotein; LDL, low-density lipoprotein; MCV, mean corpuscular volume; MCH, mean corpuscular hemoglobin; RDW, red blood cell distribution width.

### Cytokine analysis

Patient serum was analyzed for IL-12p70, IFN-γ, IL-17A, IL-2, MCP-1, IL-10, IL-8, IL-6, IFN-α, IL-1β and TNF-α using the bead based FlowCytomix Multiplex Kit (Bender MedSystems, Vienna, Austria) in accordance with the manufacturer's instructions.

### Immunohistochemistry

During surgery, 5-mm segments of the radial artery and cephalic vein were harvested for histological evaluation. The biopsies were fixed in formalin and embedded in paraffin. Sections (4 µm thick) were cut, deparaffinized in xylene (VWR International, Radnow, PA), and hydrated in a descending alcohol series. After sequential incubation with 3% hydrogen peroxide (Merck, Darmstadt, Germany) for 15 minutes at room temperature to block endogenous peroxidase, with avidin and biotin (DakoCytomation, Glostrop, Denmark) for 20 minutes at room temperature, and with Fc-receptor blocker for 30 minutes (Innovex Biosciences, Richmond, CA), the slides were incubated with primary antibodies against CD68 (IgG3, clone: PG-M1), CD45 (IgG1, clone: 2B11+PD7/26), and CD31 (All from DakoCytomation, Glostrop, Denmark) overnight at 4°C. Positive cells were detected with the Supersensitive Link Label Detection System (BioGenex, San Ramon CA). Briefly, slides were incubated with biotinylated goat anti-mouse antibodies and then with horseradish peroxidase–labeled streptavidin. Antigens were visualized with diaminobenzidine (Innovex Biosciences, Richmond, CA). The slides were counterstained with hematoxylin (Vector Laboratories, Burlingame, CA) before mounting. Positive cells and all cells in the biopsies were counted. The percentage of positive cells or grading as positive or negative was used in the subsequent analysis.

### Statistical analysis

Differences in categorical factors were determined with the chi-square test; differences in continuous factors were determined by one-way analysis of variance. Differences in continuous values between two groups were assessed with the *t* test or Mann-Whitney U test.

For univariable analysis of all factors, Cox proportional hazards models were used. All factors that met the significance criterion (*p*<0.2) were considered for inclusion in the final model; to avoid multicollinearity, these factors were analyzed with Pearson's correlation coefficient. For variables with a correlation coefficient >0.8, only one of the variables was kept in the final model; this decision was based on medical judgment. Due to the small sample size only biochemical factors were considered for inclusion in the final model, thus medication with calcium antagonists (*p* = 0.06) was excluded. Receiver operating characteristic (ROC) curves were used to establish a cutoff point between high and low value for all the significant factors in multivariable analysis. Survival curves were calculated for the various groups using the Kaplan-Meier method and compared by the log-rank test. All values are reported as mean ± standard deviation (SD) or percentages; 95% confidence intervals (CIs) are provided where appropriate. MedCalc version 12.2.1 and GraphPad Prism version 4.0c software were used for all analyses and graphs.

## Results

### Inflammatory markers are significantly higher in patients with early AVF dysfunction

Of the 68 patients in the study, 11 (16%) had early AVF dysfunction (defined as failure to mature or thrombosis within 2 months) and 23 (34%) patients had late dysfunction (3–24 months). The average time to dysfunction was 7±6 months. The AVF failure rate at 24 months was 50%; 13% were due to primary failure, consistent with previous observations [5], [15]. In the patient population with AVF survival >24 months, 6 (18%) patients had coronary disease, stroke or transient ischemic attack. Similar incident rates, 2 patients (18%), were observed in the early AVF dysfunction group. These incidents were although more prevalent in the late AVF dysfunction patient population where 15 (65%) patients were affected.

The demographic, biochemical, and clinical characteristics of the patients are shown in Table 1 and Table S1. Patients with early AVF dysfunction had higher C-reactive protein (CRP) levels than patients with late dysfunction or no dysfunction (18.6±16.8 versus 8.6±7.7 and 7.3±6.6, respectively; *p* = 0.01), a higher white blood cell count (WBC) (9.3±2.3 versus 7.2±1.9 and 7.1±1.7, *p* = 0.01), red blood cell distribution width (RDW) (16.9±2.4 versus 15.6±2.3 and 14.8±1.7, *p* = 0.03), and a higher platelet count (329±143 versus 231±80 and 257±89, *p* = 0.049). There were no significant longitudinal variations in RDW levels (Figure S1). Patients with early or late AVF dysfunction had a lower red blood cell count (RBC) (3.2±0.3 and 3.5±0.4 versus 3.7±0.6×10^12^/L, *p* = 0.048) and lower levels of serum albumin (3.2±1.1 and 3.6±0.6 versus 4.0±0.4 g/dL, *p* = 0.003). There were no differences in patient characteristics, medications, or the remaining biochemical parameters analyzed. No differences in the serum levels of IL-12p70, IFN-γ, IL-17A, IL-2, MCP-1, IL-10, IL-8, IL-6, IFN-α, IL-1β and TNF-α were found (Table S2).

### High RDW, WBC and monocyte levels are independent risk factors for AVF failure

The individual predictive values of all parameters were assessed by univariable analysis using Cox regression models (Table 2; results from parameters in Table S1 are not shown but all were *p*>0.2). Increases in CRP (HR 1.04; 95% CI 1.00 to 1.08; *p* = 0.040), WBC (1.22; 95% CI 1.00 to 1.49; *p* = 0.048), or RDW (HR 1.18; 95% CI 1.02 to 1.37; *p* = 0.029) significantly increased the risk of AVF dysfunction. The risk of AVF dysfunction was reduced by increases in serum albumin (HR 0.43; 95% CI 0.26 to 0.71; *p* = 0.001), calcium (HR 0.05; 95% CI 0.01 to 0.28; *p* = 0.001) and RBC (HR 0.46; 95% CI 0.22 to 0.96; *p* = 0.040). Of note, no significant predictive effects of calcium were retained after adjustment for albumin (HR 0.60; 95% CI 0.30 to 1.19; *p* = 0.145).

For the multivariable analyses, all potentially significant biochemical variables (*p*<0.2) in the univariable analyses were included. Hematocrit and hemoglobin were excluded from the final model because they correlated strongly with RBC (r^2^ = 0.86 and r^2^ = 0.82, respectively; both *p*<0.001). Neutrophil count was excluded because it correlated strongly with WBC (r^2^ = 0.81; *p*<0.001). The WBC (HR 1.67; 95% CI 1.24 to 2.25; *p*<0.001), number of monocytes (HR 0.02; 95% CI 0.00 to 0.21; *p* = 0.001), and RDW (HR 1.44; 95% CI 1.17 to 1.78; *p*<0.001) were significant independent predictors of AVF function after adjustment for RBC, albumin, albumin-corrected calcium and CRP. To exclude the possibility that the risk increase reflected by RDW was confounded by anemia, we analyzed additional models in which RBC was replaced with hemoglobin or hematocrit. The WBC, monocyte numbers, and RDW remained significant independent predictors of AVF function in these models (data not shown).

**Figure 1:**
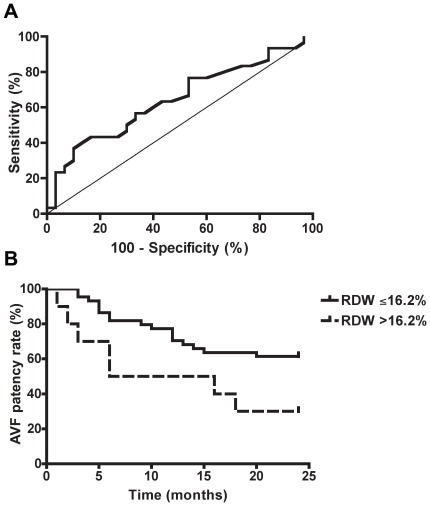
RDW is a predictor of AVF patency. Receiver operating characteristic curve analysis for RDW showed an area under the curve of 0.644 (CI 0.51 to 0.76; *p* = 0.046) (A). RDW values in the highest quartile (>16.2%) were associated with a significantly reduced AVF patency rate at 24 months after surgery (*p* = 0.036). The Kaplan-Meier curves were compared with the log-rank test (B).

Receiver operating characteristic (ROC) curve analysis was performed for WBC, monocyte count and RDW to determine appropriate cutoff values. Significant differences in the distribution were only found for RDW (area under the curve 0.644; CI 0.51 to 0.76; *p* = 0.046) (Figure 1A). On the basis of ROC curve analysis the upper quartile of RDW values (>16.2% versus ≤16.2%) was found to be the best cutoff value for predicting AVF failure. In the patients with RDW values in the highest quartile, Kaplan-Meier plots demonstrated an increased frequency of AVF failure (*p* = 0.036, log-rank test) (Figure 1B). At the end of the observation period, the percentage of patients with a functioning AVF was much lower in patients with RDW values in the highest quartile than in patients with RDW values in the three lower quartiles combined (21% versus 59%, and 30% versus 61% if primary failures are excluded).

The WBC and RDW correlated negatively with albumin (r^2^ = −0.29, *p* = 0.039 and r^2^ = −0.31, *p* = 0.025, respectively). RDW correlated positively with CRP (r^2^ = 0.30; *p* = 0.028) but showed no correlation with hematocrit, hemoglobin, or RBC. Monocyte numbers, which also independently predicted AVF dysfunction, correlated positively with RBC (r^2^ = 0.34, *p* = 0.010), hematocrit (r^2^ = 0.31, *p* = 0.019), and hemoglobin (r^2^ = 0.28, *p* = 0.031). Although statistically significant, all theses correlations were weak.

### Inflammatory cells are frequently found in the cephalic vein and radial artery

**Figure 2:**
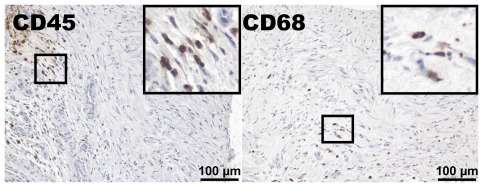
Inflammatory cells are present in the arterial and venous vessel wall. Leukocytes, defined as CD45-positive cells, were found in the radial artery or cephalic vein or both in 39% of the patients. Monocytes/macrophages, defined as CD68-positive cells, were found in the vessel wall in 37% of the patients. Both cell types were most frequently located in the arterial and venous intima and media. The larger squares are magnifications of the smaller ones.

**Figure 3:**
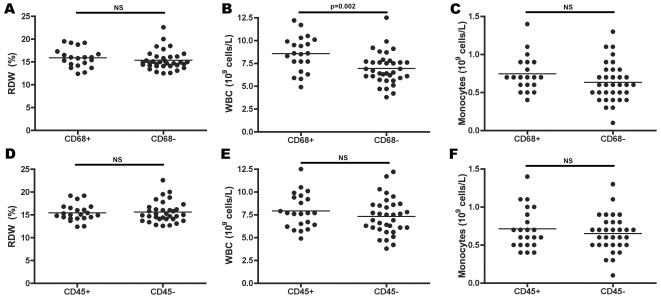
Association between presence of inflammatory cells in the vessel wall and serum parameters. A–C, Patients in whom CD68-positive cells were found in the radial artery or cephalic vein wall had significantly higher WBCs (*p* = 0.002) (B), but there were no significant differences in RDW (A) or the number of monocytes (C). D–F, No significant differences in levels of RDW (D), WBC (E), or number of monocytes (F) were found in patients whose radial artery or cephalic vein wall contained CD45-positive cells.

Inflammatory cells in the cephalic vein are found in 25% of patients at the time of AVF construction [16], and are frequently found in failed AVFs [17]. Since RDW may reflect an inflammatory state that potentially could reflect the increased risk associated with high RDW, we evaluated the radial artery and cephalic vein biopsies for inflammatory cells. CD68-positive cells (monocytes/macrophages) were found in 37% of the patients, and leukocytes (CD45-positive cells) were found in 39% of the patients; both types of cells were located predominantly in the arterial and venous intima and media (Figure 2). No significant differences in the number of CD68- or CD45-positive cells in the arteries or veins were observed between the three patient subpopulations divided according to AVF patency time (data not shown). The patients in whom CD68-positive cells were found in the vessel biopsies had significantly higher WBCs (*p* = 0.002) (Figure 3B) but no significant differences in RDW or monocyte numbers (Figure 3). No significant differences in WBC, RDW, or monocyte numbers were found in patients whose arteries/veins were infiltrated by CD45-positive cells (Figure 3).

## Discussion

AVF dysfunction remains a major cause of morbidity in hemodialysis patients and leads to significant health care costs. This prospective study showed that WBC, monocyte count, and RDW are significant independent predictors of AVF dysfunction. Importantly, the magnitude of the increased risk for AVF failure associated with increases in WBC and RDW was clinically relevant. A one-unit increase in WBC or RDW increased the risk of AVF failure by 67% and 44%, respectively, and RDW>16.2% was associated with a significantly reduced AVF patency rate 24 months after surgery.

RDW is a measurement of anisocytosis and was initially constructed to aid in the evaluation of anemia. It is calculated as the standard deviation of the red blood cell volume divided by the mean corpuscular volume and is routinely reported by automated laboratory blood count equipment [18]. RDW has emerged as a strong independent predictive factor for mortality in patients with chronic and acute heart failure [19]–[21], acute coronary syndromes [22], and pulmonary hypertension [23]. Furthermore, in a large study, RDW was related to the risk of death and cardiovascular events in patients without symptomatic heart failure who had suffered a myocardial infarction [24]. The usefulness of RDW as a predictor of thromboembolic conditions has been sparsely evaluated; however, patients with an elevated RDW are more likely to have had a stroke (OR 1.71; 95% CI 1.20–2.45) [25]. RDW is readily available and affordable, which increases its value as a prognostic marker.

The mechanisms by which elevation in RDW contributes to an increase in mortality risk and the risk for cardiovascular events are unknown. It has been suggested that RDW reflects ineffective red blood cell production, inflammation, impaired renal function, and malnutrition [20]. Indeed, RDW is increased in patients with iron, folate, or vitamin B_12_ deficiency [18]. The risk for AVF failure associated with increasing RDW is likely not due to anemia, as neither hematocrit, hemoglobin, iron, transferrin nor ferritin levels affected AVF failure risk in univariable analysis or correlated with RDW. In the multivariable analysis, RDW remained a significant risk factor after adjustment for RBC/hematocrit/hemoglobin and other factors, making it unlikely that our findings were confounded by anemia. Information on folate or vitamin B_12_ deficiencies in our patients were not available. No risk was associated with mean corpuscular volume (MCV), which is affected in these deficiencies (Table 2), and the MCV values did not differ significantly when the patients were divided according to the time to AVF failure (Table 1). As inflammation and iron deficiency tend to result in decreased MCV while vitamin B_12_ and B_9_ deficiencies are associated with increased MCV a combination of these causes could affect RDW while MCV would remain normal. Therefore, we cannot fully exclude the possibility that folate or vitamin B_12_ deficiencies affected RDW in our study.

RDW potentially reflects an underlying inflammatory state that leads to AVF failure. Besides RDW, WBC was an independent prognostic marker of AVF failure, and RDW correlated positively with CRP. These findings are consistent with previous reports of positive correlations between RDW and the inflammatory markers IL-6, CRP, and erythrocyte sedimentation rate [20], [26]. Several of these markers have been associated with AVF thrombosis [13], [14], but this is to our knowledge the first time RDW has been identified as a prognostic marker for AVF failure. The relationship between RDW and inflammation is still equivocal. Weak but significant positive correlation with CRP, as found in our study, has also been identified in patients with coronary disease [27]. Interestingly, the presence of inflammatory cells in the artery/vein at the time of surgery was not associated with an increase in RDW or monocyte levels. One explanation may be that RDW is a very early marker of inflammation that precedes inflammatory cell infiltration. An elevated RDW may also reflect IL-6-mediated increases in vascular smooth muscle cell migration and proliferation [28], [29], which potentially promote AVF failure.

Correlations between RDW and malnutrition may in part explain the association between RDW and mortality in heart failure [20]. Malnutrition has also been identified as a risk factor for AVF failure [10]. In our study, no significant differences in body mass index (BMI), cholesterol or transferrin levels were observed between the patient groups. Albumin was lower in the early AVF failure group but as it is affected by numerous factors other than nutrition intake it is not a useful marker for malnutrition [30]. Although we cannot fully exclude effects of potential malnutrition on RDW levels it seems unlikely that this is the main mechanistic link between high RDW and AVF failure in our study.

Red blood cell deformability, aggregability and adherence to the blood vessel wall endothelium can affect blood flow and contribute to thrombosis. Increases in red blood cell aggregation have been associated with thrombosis in for example diabetes, coronary heart disease and hypertension [31], [32]. High RDW can reflect the effects of erythroid or nonerythroid pathologies on the red blood cell population, potentially associated with increased RBC deformability, aggregability and/or adherence to the vessel wall, contributing to thrombosis formation and subsequent AVF failure.

Importantly, RDW seems to be a better predictor of AVF failure then inflammatory markers such as CRP and WBC. CRP was not a significant risk factor in multivariable analysis and the significant correlation found between CRP and RDW was weak (r^2^ = 0.30). WBC, although being a significant risk factor in multivariable analysis, was not significant in ROC curve analysis indicating that as a laboratory test it has not the ability to distinguish between the AVF failure and non-failure groups. RDW may represent an integrative measure of multiple pathologic processes, such as inflammation, nutritional defects and red blood cell aggregation, explaining its strong association with AVF dysfunction.

The strengths of our study are its prospective design, the comprehensive biochemical data collected at the time of surgery, and the availability of vessel biopsies. A potential limitation is that patients were enrolled consecutively at a single center, so the patient population was relatively small and heterogeneous. In summary, we identified RDW, WBC, and the number of monocytes as independent predictors of AVF failure. Further studies in a larger patient cohort and involving several centers, including further potential factors affecting RDW, are needed to verify the prognostic value of RDW and to provide insight into the mechanistic link between RDW and AVF failure.

## Supporting Information

Figure S1 Longitudinal variations in RDW. No statistically significant longitudinal variations in RDW levels were observed.
(TIF)

Table S1 Patient characteristics.
(DOCX)

Table S2 Patient cytokine levels.
(DOCX)
