# Supplementary figures and images for: A High Red Blood Cell Distribution Width Predicts Failure of Arteriovenous Fistula
Source: PLoS One. 2012 May 4;7(5):e36482. doi: 10.1371/journal.pone.0036482 (PMC3344886; doi:10.1371/journal.pone.0036482)

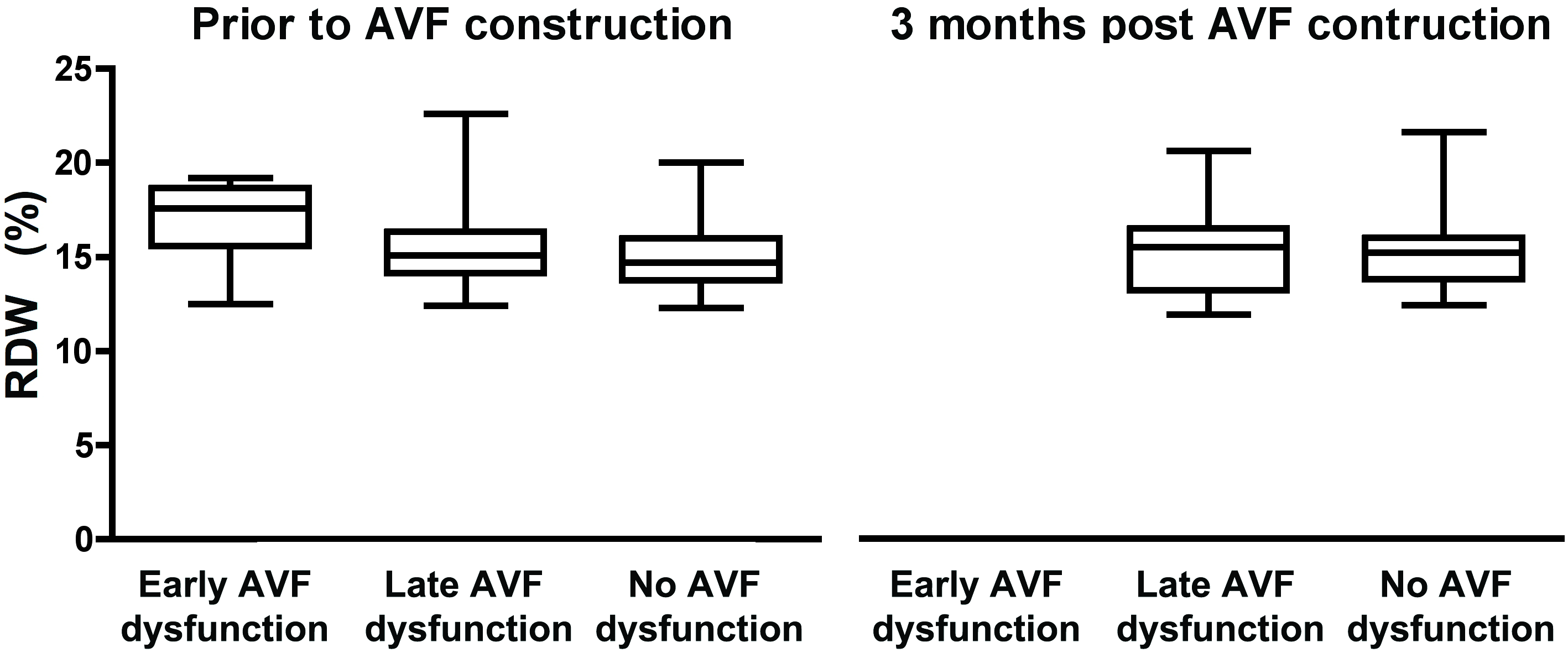

Supplement: Figure S1 — Longitudinal variations in RDW. No statistically significant longitudinal variations in RDW levels were observed. (TIF) [file pone.0036482.s001.tif]
