# Supplementary material for: A High Red Blood Cell Distribution Width Predicts Failure of Arteriovenous Fistula
Source: PLoS One. 2012 May 4;7(5):e36482. doi: 10.1371/journal.pone.0036482 (PMC3344886; doi:10.1371/journal.pone.0036482)
Supplement: Table S1 — Patient characteristics. (DOCX) [file pone.0036482.s002.docx]

**Supporting Table 1. Patient characteristics**

| Patient characteristics | Early AVF dysfunction  (n = 11) | Late AVF dysfunction (n=23) | No AVF dysfunction (n=34) | *p* | All patients  (n = 68) |
| --- | --- | --- | --- | --- | --- |
| Body mass index (kg/m^2^, ± SD) | 24.9 ± 3.4 | 25.3 ± 6.1 | 25.9 ± 3.9 | 0.83 | 25.6 ± 4.6 |
| Medications |  |  |  |  |  |
| Erythropoietin (units, ± SD) | 3188 ± 2304 | 4050 ± 2266 | 3080 ± 1949 | 0.47 | 3361 ± 2060 |
| Fe (mg, ± SD) | 50.0 ± 50.0 | 75.0 ± 43.3 | 63.1 ± 38.6 | 0.62 | 65.5 ± 40.4 |
| Biochemical parameters |  |  |  |  |  |
| Glucose (mg/dL, ± SD) | 89.6 ± 19.9 | 102.4 ± 34.6 | 104.6 ± 34.9 | 0.57 | 102.0 ± 33.2 |
| Total cholesterol (mg/dL, ± SD) | 189.3 ± 64.5 | 198.3 ± 75.0 | 185.2 ± 58.2 | 0.79 | 190.2 ± 64.2 |
| Urea (mg/dL, ± SD) | 99.9 ± 39.6 | 111.9 ± 53.0 | 114.4 ± 43.8 | 0.74 | 111.5 ± 46.3 |
| INR (± SD) | 0.99 ± 0.05 | 1.0 ± 0.10 | 0.97 ± 0.06 | 0.38 | 0.99 ± 0.08 |
| Fibrinogen (mg/dL, ± SD) | 768.9 ± 252.6 | 600.6 ± 180.8 | 625.7 ± 177.1 | 0.13 | 635.4 ± 192.8 |
| D-dimer (μg/dL, ± SD) | 243.4 ± 80.5 | 294.7 ± 188.6 | 319.6 ± 214.2 | 0.64 | 301 ± 191.9 |
| APTT (seconds, ± SD) | 33.9 ± 6.8 | 31.8 ± 4.3 | 31.2 ± 4.4 | 0.39 | 31.8 ± 4.7 |
| Antithrombin III (μg/mL*, ±* SD) | 89.4 ± 20.1 | 91.5 ± 21.3 | 97.0 ± 14.9 | 0.46 | 93.9 ± 18.1 |
| Potassium (mmol/L, ± SD) | 4.7 ± 0.9 | 4.6 ± 0.6 | 4.8 ± 0.7 | 0.57 | 4.7 ± 0.7 |
| Sodium (mmol/L, ± SD) | 136.8 ± 3.8 | 136.6 ± 5.3 | 137.3 ± 4.5 | 0.85 | 137.0 ± 4.7 |
| Phosphate (mg/dL, ± SD) | 4.2 ± 0.9 | 4.6 ± 1.3 | 4.6 ± 1.1 | 0.69 | 4.6 ± 1.2 |
| MCHC (%, ± SD) | 33.7 ± 1.1 | 33.8 ± 1.4 | 33.4 ± 1.4 | 0.50 | 33.6 ± 1.3 |
| Mean platelet volume (fL, ± SD) | 7.44 ± 0.91 | 8.57 ± 1.44 | 8.50 ± 1.22 | 0.08 | 8.39 ± 1.31 |
| Creatine kinase (μg/L, ± SD) | 29.9 ± 21.6 | 23.7 ± 10.2 | 22.3 ± 12.6 | 0.40 | 23.7 ± 13.2 |

INR, international normalized ratio; APTT, activated partial thromboplastin time; MCHC, mean corpuscular hemoglobin concentration.
