# Supplementary material for: A High Red Blood Cell Distribution Width Predicts Failure of Arteriovenous Fistula
Source: PLoS One. 2012 May 4;7(5):e36482. doi: 10.1371/journal.pone.0036482 (PMC3344886; doi:10.1371/journal.pone.0036482)
Supplement: Table S2 — Patient cytokine levels. (DOCX) [file pone.0036482.s003.docx]

**Supporting Table 2. Patient cytokine levels**

| Cytokine (pg/ml) | Early AVF dysfunction (n=8) | Late AVF dysfunction (n=20) | No AVF dysfunction (n=33) | P value* |
| --- | --- | --- | --- | --- |
| IL-12p70 | 108.5 (0 - 449.6) | 66.4 (0 – 359.4) | 79.8 (0 – 862.6) | 0.91 |
| IFN-γ | 88.9 (0 - 379.3) | 63.4 (0 – 478.7) | 72.1 (0 - 663.5) | 0.58 |
| IL-17A | 37.8 (0 – 291.0) | 24.7 (0 – 288.7) | 23.1 (0 – 61.9) | 0.88 |
| IL-2 | 153.9 (0 – 264.5) | 147.6 (0 – 586.9) | 170.9 (0 – 1408.9) | 0.94 |
| MCP-1 | 975.2 (0 – 1867.0) | 1056.2 (634 – 1533.1) | 1126.8 (90 – 1613.0) | 0.39 |
| IL-10 | 27.6 (0 – 192.9) | 10.0 (0 – 110.9) | 15.5 (0 – 383.4) | 0.83 |
| IL-8 | 41.0 (0 – 139.9) | 35.6 (0 – 345.6) | 53.4 (0 – 671.7) | 0.71 |
| IL-6 | 5.3 (0 – 29.3) | 3.0 (0 – 60.3) | 0.4 (0 – 5.6) | 0.25 |
| IFN-α | 282.2 (0 – 885.0) | 245.9 (0 – 1094.0) | 229.2 (0 – 1344.8) | 0.97 |
| IL-1β | 20.9 (0 – 162.0) | 12.9 (0 – 204.8) | 9.4 (0 – 242.4) | 0.86 |
| TNF-α | 25.9 (0 – 176.9) | 11.4 (0 – 144.1) | 12.2 (0 – 273.5) | 0.63 |

Values are expressed as mean with minimum – maximum range.

* Kruskal Wallis test was used
